# 2-Chloro-3-hydroxy­methyl-6-methoxy­quinoline

**DOI:** 10.1107/S1600536809054051

**Published:** 2009-12-19

**Authors:** F. Nawaz Khan, S. Mohana Roopan, Venkatesha R. Hathwar, Seik Weng Ng

**Affiliations:** a Chemistry Division, School of Science and Humanities, VIT University, Vellore 632 014, Tamil Nadu, India; b Solid State and Structural Chemistry Unit, Indian Institute of Science, Bangalore 560 012, Karnataka, India; c Department of Chemistry, University of Malaya, 50603 Kuala Lumpur, Malaysia

## Abstract

All the non-H atoms of the title compound, C_11_H_10_ClNO_2_, are roughly coplanar (r.m.s. deviation = 0.058 Å). In the crystal, adjacent mol­ecules are linked by an O—H⋯N hydrogen bond, generating chains running along the *a* axis.

## Related literature

Substituted quinoline-3-carbaldehydes are inter­mediates for annelation and functional group modification; for a review of the synthesis of quinolines by the Vilsmeier–Haack reaction, see: Meth-Cohn (1993 ▶).
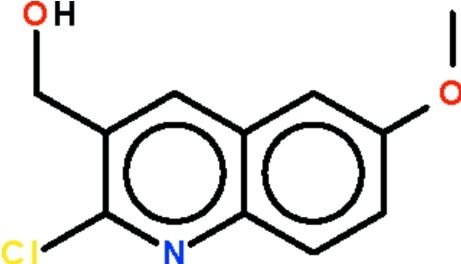


## Experimental

### 

#### Crystal data

C_11_H_10_ClNO_2_
*M* *_r_* = 223.65
Monoclinic, 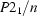

*a* = 6.9738 (3) Å
*b* = 21.4668 (9) Å
*c* = 7.3479 (4) Å
β = 108.220 (5)°
*V* = 1044.87 (8) Å^3^
*Z* = 4
Mo *K*α radiation
μ = 0.34 mm^−1^
*T* = 293 K
0.28 × 0.21 × 0.20 mm

#### Data collection

Bruker SMART area-detector diffractometer
Absorption correction: multi-scan (*SADABS*; Sheldrick, 1996 ▶) *T*_min_ = 0.910, *T*_max_ = 0.935
11517 measured reflections
2348 independent reflections
1487 reflections with *I* > 2σ(*I*)
*R*_int_ = 0.035

#### Refinement

*R*[*F* ^2^ > 2σ(*F* ^2^)] = 0.041
*wR*(*F* ^2^) = 0.108
*S* = 0.97
2348 reflections
138 parameters
H-atom parameters constrained
Δρ_max_ = 0.21 e Å^−3^
Δρ_min_ = −0.25 e Å^−3^

### 

Data collection: *SMART* (Bruker, 2004 ▶); cell refinement: *SAINT* (Bruker, 2004 ▶); data reduction: *SAINT*; program(s) used to solve structure: *SHELXS97* (Sheldrick, 2008 ▶); program(s) used to refine structure: *SHELXL97* (Sheldrick, 2008 ▶); molecular graphics: *X-SEED* (Barbour, 2001 ▶); software used to prepare material for publication: *publCIF* (Westrip, 2010 ▶).

## Supplementary Material

Crystal structure: contains datablocks global, I. DOI: 10.1107/S1600536809054051/bt5140sup1.cif

Structure factors: contains datablocks I. DOI: 10.1107/S1600536809054051/bt5140Isup2.hkl

Additional supplementary materials:  crystallographic information; 3D view; checkCIF report

## Figures and Tables

**Table 1:** Hydrogen-bond geometry (Å, °)

*D*—H⋯*A*	*D*—H	H⋯*A*	*D*⋯*A*	*D*—H⋯*A*
O1—H1⋯N1^i^	0.82	2.16	2.913 (2)	153

Symmetry code: (i) 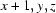.

**Fig. 1.**
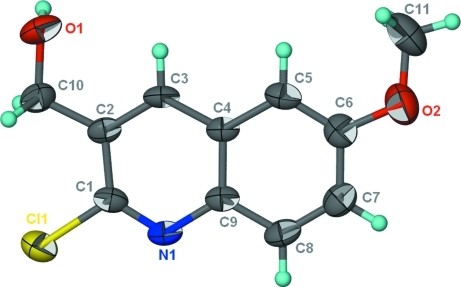
Anisotropic displacement ellipsoid plot (Barbour, 2001) of C11H10ClNO2 at the 50% probability level; hydrogen atoms are drawn as spheres of arbitrary radius.

## supplementary crystallographic information

### Experimental

2-Chloro-8-methoxyquinoline-3-carbaldehyde (220 mg, 1 mmol), sodium borohydride (38 mg, 1 mmol) and a catalytic amount of montmorillonite K-10 were placed in a beaker. The contents were irradiated at 500 W for 5 min. The product was dissolved in ethyl acetate and the residue removed by filtration. The filtrate was subjected to column chromatography on silica, and ethyl acetate/petroleum ether was used as the eluant. The solvent was evaporated and the residue recrystallized from chloroform to give colorless crystals.

### Refinement

Hydrogen atoms were placed in calculated positions (C–H 0.93–0.97, O–H 0.82 Å) and were included in the refinement in the riding model approximation, with *U*(H) set to 1.2–1.5*U*(C,*O*).

### Crystal data

**Table:**

C_11_H_10_ClNO_2_	*F*(000) = 464
*M**_r_* = 223.65	*D*_x_ = 1.422 Mg m^−^^3^
Monoclinic, *P*2_1_/*n*	Mo *K*α radiation, λ = 0.71073 Å
Hall symbol: -P 2yn	Cell parameters from 1941 reflections
*a* = 6.9738 (3) Å	θ = 3.1–25.5°
*b* = 21.4668 (9) Å	µ = 0.34 mm^−^^1^
*c* = 7.3479 (4) Å	*T* = 293 K
β = 108.220 (5)°	Block, colorless
*V* = 1044.87 (8) Å^3^	0.28 × 0.21 × 0.20 mm
*Z* = 4	

### Data collection

**Table:**

Bruker SMART area-detector diffractometer	2348 independent reflections
Radiation source: fine-focus sealed tube	1487 reflections with *I* > 2σ(*I*)
graphite	*R*_int_ = 0.035
φ and ω scans	θ_max_ = 27.5°, θ_min_ = 3.1°
Absorption correction: multi-scan (*SADABS*; Sheldrick, 1996)	*h* = −9→8
*T*_min_ = 0.910, *T*_max_ = 0.935	*k* = −26→27
11517 measured reflections	*l* = −9→9

### Refinement

**Table:**

Refinement on *F*^2^	Primary atom site location: structure-invariant direct methods
Least-squares matrix: full	Secondary atom site location: difference Fourier map
*R*[*F*^2^ > 2σ(*F*^2^)] = 0.041	Hydrogen site location: inferred from neighbouring sites
*wR*(*F*^2^) = 0.108	H-atom parameters constrained
*S* = 0.97	*w* = 1/[σ^2^(*F*_o_^2^) + (0.058*P*)^2^] where *P* = (*F*_o_^2^ + 2*F*_c_^2^)/3
2348 reflections	(Δ/σ)_max_ = 0.001
138 parameters	Δρ_max_ = 0.21 e Å^−^^3^
0 restraints	Δρ_min_ = −0.25 e Å^−^^3^

### Fractional atomic coordinates and isotropic or equivalent isotropic displacement parameters (Å^2^)

**Table:**

	*x*	*y*	*z*	*U*_iso_*/*U*_eq_	
Cl1	0.33210 (8)	0.65210 (2)	0.10064 (7)	0.0648 (2)	
O1	0.97027 (18)	0.59162 (7)	0.33602 (19)	0.0672 (4)	
H1	1.0200	0.5790	0.2554	0.101*	
O2	0.4314 (2)	0.29302 (7)	0.3876 (2)	0.0766 (5)	
N1	0.2607 (2)	0.53770 (7)	0.16969 (18)	0.0446 (4)	
C1	0.4061 (3)	0.57605 (8)	0.1763 (2)	0.0430 (4)	
C2	0.6156 (2)	0.56246 (8)	0.2401 (2)	0.0408 (4)	
C3	0.6650 (2)	0.50291 (8)	0.2994 (2)	0.0437 (4)	
H3	0.8004	0.4916	0.3461	0.052*	
C4	0.5149 (2)	0.45775 (8)	0.2917 (2)	0.0390 (4)	
C5	0.5600 (3)	0.39542 (9)	0.3483 (2)	0.0494 (5)	
H5	0.6935	0.3821	0.3926	0.059*	
C6	0.4070 (3)	0.35449 (9)	0.3379 (2)	0.0526 (5)	
C7	0.2053 (3)	0.37443 (9)	0.2741 (2)	0.0546 (5)	
H7	0.1027	0.3463	0.2698	0.065*	
C8	0.1583 (3)	0.43397 (9)	0.2189 (2)	0.0507 (5)	
H8	0.0238	0.4463	0.1758	0.061*	
C9	0.3120 (2)	0.47761 (8)	0.2261 (2)	0.0405 (4)	
C10	0.7702 (3)	0.61178 (9)	0.2442 (3)	0.0528 (5)	
H10A	0.7575	0.6240	0.1138	0.063*	
H10B	0.7430	0.6482	0.3103	0.063*	
C11	0.6296 (4)	0.26820 (11)	0.4324 (4)	0.0912 (8)	
H11A	0.6799	0.2747	0.3263	0.137*	
H11B	0.6264	0.2244	0.4573	0.137*	
H11C	0.7163	0.2887	0.5439	0.137*	

### Atomic displacement parameters (Å^2^)

**Table:**

	*U*^11^	*U*^22^	*U*^33^	*U*^12^	*U*^13^	*U*^23^
Cl1	0.0574 (3)	0.0603 (3)	0.0736 (4)	0.0151 (2)	0.0158 (3)	0.0111 (3)
O1	0.0365 (8)	0.0931 (11)	0.0762 (9)	−0.0030 (7)	0.0235 (7)	−0.0079 (8)
O2	0.0887 (12)	0.0522 (9)	0.0807 (10)	0.0010 (8)	0.0147 (8)	0.0016 (7)
N1	0.0306 (8)	0.0591 (9)	0.0412 (8)	0.0057 (7)	0.0069 (6)	−0.0035 (7)
C1	0.0389 (10)	0.0539 (10)	0.0365 (9)	0.0122 (8)	0.0123 (8)	−0.0006 (8)
C2	0.0347 (9)	0.0573 (11)	0.0341 (8)	0.0033 (8)	0.0161 (7)	−0.0036 (8)
C3	0.0277 (9)	0.0628 (11)	0.0403 (9)	0.0106 (8)	0.0103 (7)	−0.0021 (8)
C4	0.0328 (9)	0.0524 (10)	0.0307 (8)	0.0059 (8)	0.0083 (7)	−0.0051 (7)
C5	0.0420 (11)	0.0593 (12)	0.0428 (10)	0.0127 (9)	0.0076 (8)	−0.0032 (9)
C6	0.0589 (13)	0.0534 (12)	0.0427 (10)	−0.0005 (9)	0.0119 (9)	−0.0073 (9)
C7	0.0504 (12)	0.0631 (13)	0.0476 (10)	−0.0115 (10)	0.0115 (9)	−0.0095 (9)
C8	0.0334 (10)	0.0698 (13)	0.0443 (10)	−0.0022 (9)	0.0056 (8)	−0.0093 (9)
C9	0.0334 (9)	0.0559 (11)	0.0304 (8)	0.0047 (8)	0.0070 (7)	−0.0069 (8)
C10	0.0433 (11)	0.0653 (12)	0.0547 (11)	0.0001 (9)	0.0223 (9)	0.0013 (10)
C11	0.113 (2)	0.0600 (14)	0.1016 (18)	0.0275 (14)	0.0342 (16)	0.0121 (13)

### Geometric parameters (Å, °)

**Table:**

Cl1—C1	1.7496 (18)	C4—C9	1.411 (2)
O1—C10	1.414 (2)	C5—C6	1.366 (2)
O1—H1	0.8200	C5—H5	0.9300
O2—C6	1.366 (2)	C6—C7	1.403 (3)
O2—C11	1.421 (3)	C7—C8	1.350 (3)
N1—C1	1.295 (2)	C7—H7	0.9300
N1—C9	1.368 (2)	C8—C9	1.412 (2)
C1—C2	1.418 (2)	C8—H8	0.9300
C2—C3	1.360 (2)	C10—H10A	0.9700
C2—C10	1.505 (2)	C10—H10B	0.9700
C3—C4	1.415 (2)	C11—H11A	0.9600
C3—H3	0.9300	C11—H11B	0.9600
C4—C5	1.407 (2)	C11—H11C	0.9600
			
C10—O1—H1	109.5	C8—C7—C6	120.86 (18)
C6—O2—C11	117.08 (18)	C8—C7—H7	119.6
C1—N1—C9	117.42 (14)	C6—C7—H7	119.6
N1—C1—C2	126.53 (16)	C7—C8—C9	120.44 (17)
N1—C1—Cl1	115.57 (13)	C7—C8—H8	119.8
C2—C1—Cl1	117.90 (14)	C9—C8—H8	119.8
C3—C2—C1	115.52 (16)	N1—C9—C4	121.87 (16)
C3—C2—C10	123.17 (16)	N1—C9—C8	119.38 (15)
C1—C2—C10	121.30 (16)	C4—C9—C8	118.75 (16)
C2—C3—C4	121.42 (16)	O1—C10—C2	112.82 (16)
C2—C3—H3	119.3	O1—C10—H10A	109.0
C4—C3—H3	119.3	C2—C10—H10A	109.0
C5—C4—C9	119.71 (16)	O1—C10—H10B	109.0
C5—C4—C3	123.07 (16)	C2—C10—H10B	109.0
C9—C4—C3	117.21 (15)	H10A—C10—H10B	107.8
C6—C5—C4	119.79 (17)	O2—C11—H11A	109.5
C6—C5—H5	120.1	O2—C11—H11B	109.5
C4—C5—H5	120.1	H11A—C11—H11B	109.5
O2—C6—C5	125.23 (18)	O2—C11—H11C	109.5
O2—C6—C7	114.34 (18)	H11A—C11—H11C	109.5
C5—C6—C7	120.43 (18)	H11B—C11—H11C	109.5
			
C9—N1—C1—C2	−1.4 (2)	C4—C5—C6—C7	−1.1 (3)
C9—N1—C1—Cl1	179.45 (10)	O2—C6—C7—C8	−179.55 (16)
N1—C1—C2—C3	0.1 (2)	C5—C6—C7—C8	1.1 (3)
Cl1—C1—C2—C3	179.24 (12)	C6—C7—C8—C9	−0.6 (3)
N1—C1—C2—C10	−178.78 (16)	C1—N1—C9—C4	0.9 (2)
Cl1—C1—C2—C10	0.4 (2)	C1—N1—C9—C8	−179.17 (15)
C1—C2—C3—C4	1.7 (2)	C5—C4—C9—N1	179.93 (14)
C10—C2—C3—C4	−179.43 (15)	C3—C4—C9—N1	0.8 (2)
C2—C3—C4—C5	178.76 (15)	C5—C4—C9—C8	0.0 (2)
C2—C3—C4—C9	−2.1 (2)	C3—C4—C9—C8	−179.16 (14)
C9—C4—C5—C6	0.5 (2)	C7—C8—C9—N1	−179.89 (15)
C3—C4—C5—C6	179.60 (16)	C7—C8—C9—C4	0.1 (2)
C11—O2—C6—C5	−7.7 (3)	C3—C2—C10—O1	−6.8 (2)
C11—O2—C6—C7	173.00 (17)	C1—C2—C10—O1	171.94 (14)
C4—C5—C6—O2	179.68 (15)		

### Hydrogen-bond geometry (Å, °)

**Table:**

*D*—H···*A*	*D*—H	H···*A*	*D*···*A*	*D*—H···*A*
O1—H1···N1^i^	0.82	2.16	2.913 (2)	153

Symmetry codes: (i) *x*+1, *y*, *z*.
